# Harnessing the Potential of Real-World Evidence in the Treatment of Colorectal Cancer: Where Do We Stand?

**DOI:** 10.1007/s11864-024-01186-4

**Published:** 2024-02-17

**Authors:** Sietske C. M. W. van Nassau, Guus M. Bol, Frederieke H. van der Baan, Jeanine M. L. Roodhart, Geraldine R. Vink, Cornelis J. A. Punt, Anne M. May, Miriam Koopman, Jeroen W. G. Derksen

**Affiliations:** 1 Department of Medical Oncology, University Medical Center Utrecht, Utrecht University, Heidelberglaan 100, PO Box 85500, Utrecht, 3584 CX The Netherlands; 2 Department of Epidemiology & Health Economics, Julius Center for Health Sciences and Primary Care, University Medical Center Utrecht, Utrecht, The Netherlands; 3 Department of Research and Development, Netherlands Comprehensive Cancer Organisation (IKNL), Utrecht, The Netherlands

**Keywords:** Real-world data, Real-world evidence, Oncology, Colorectal cancer, Population-based, Efficacy-effectiveness gap

## Abstract

Treatment guidelines for colorectal cancer (CRC) are primarily based on the results of randomized clinical trials (RCTs), the gold standard methodology to evaluate safety and efficacy of oncological treatments. However, generalizability of trial results is often limited due to stringent eligibility criteria, underrepresentation of specific populations, and more heterogeneity in clinical practice. This may result in an efficacy-effectiveness gap and uncertainty regarding meaningful benefit versus treatment harm. Meanwhile, conduct of traditional RCTs has become increasingly challenging due to identification of a growing number of (small) molecular subtypes. These challenges—combined with the digitalization of health records—have led to growing interest in use of real-world data (RWD) to complement evidence from RCTs. RWD is used to evaluate epidemiological trends, quality of care, treatment effectiveness, long-term (rare) safety, and quality of life (QoL) measures. In addition, RWD is increasingly considered in decision-making by clinicians, regulators, and payers. In this narrative review, we elaborate on these applications in CRC, and provide illustrative examples. As long as the quality of RWD is safeguarded, ongoing developments, such as common data models, federated learning, and predictive modelling, will further unfold its potential. First, whenever possible, we recommend conducting pragmatic trials, such as registry-based RCTs, to optimize generalizability and answer clinical questions that are not addressed in registrational trials. Second, we argue that marketing approval should be conditional for patients who would have been ineligible for the registrational trial, awaiting planned (non) randomized evaluation of outcomes in the real world. Third, high-quality effectiveness results should be incorporated in treatment guidelines to aid in patient counseling. We believe that a coordinated effort from all stakeholders is essential to improve the quality of RWD, create a learning healthcare system with optimal use of trials and real-world evidence (RWE), and ultimately ensure personalized care for every CRC patient.

## Introduction

**Fig. 1:**
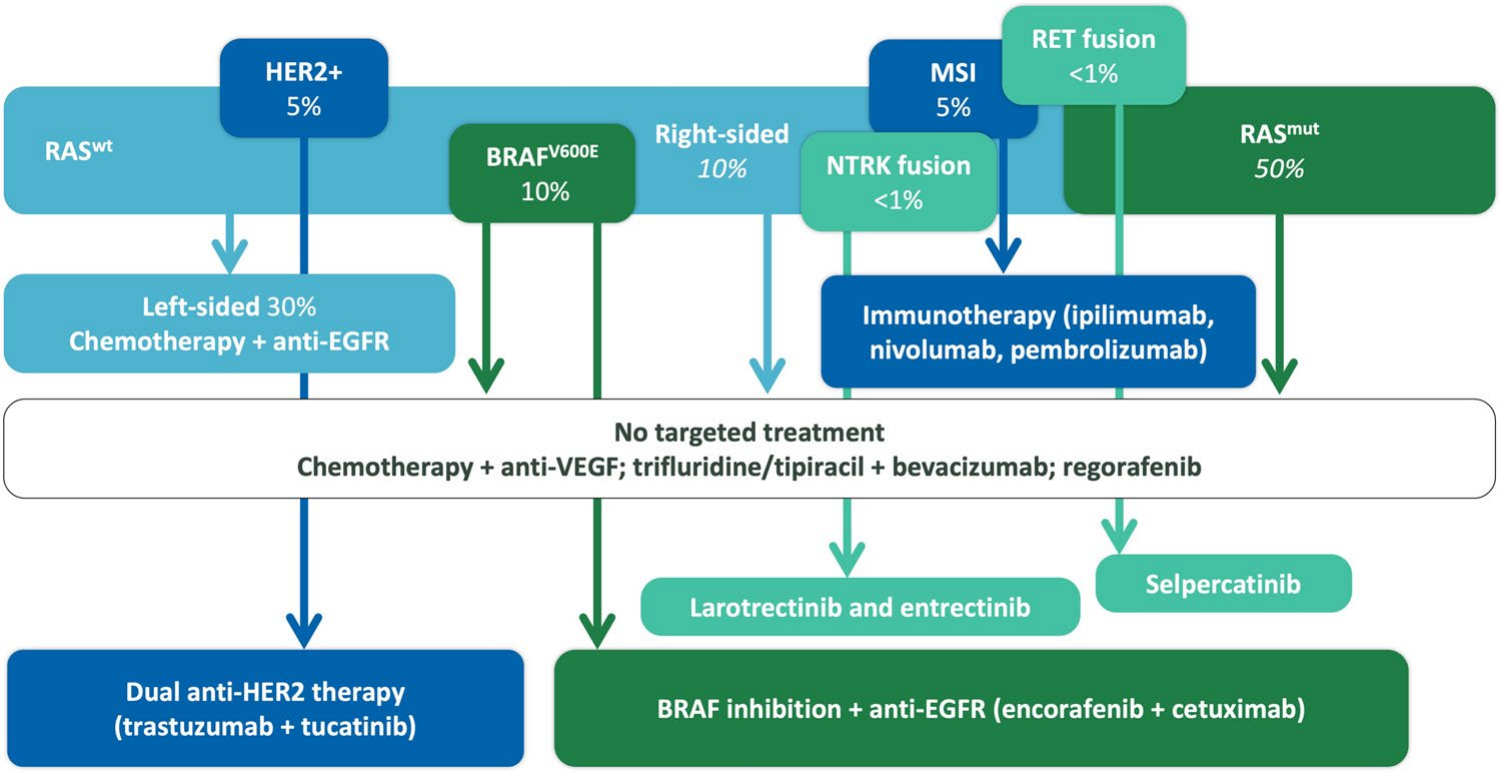
Landscape of molecularly targeted treatments* for metastatic CRC. Adapted from Punt CJA, Koopman M, and Vermeulen L. *Nat Rev Clin Oncol 14, 235–246 (2017)* [13].*Limited to FDA- and/or EMA-approved treatments. MSI, microsatellite instability; mut, mutant; wt, wild-type.

Most novel therapies become available following a successful prospective phase III RCT, the standard approach to assess treatment efficacy and safety. Although the randomized design optimizes internal validity, generalizability of oncological RCT results can be limited [1]. Most landmark trials use stringent eligibility criteria excluding a large portion of patients who will be treated in clinical practice, such as patients with multiple comorbidities, brain metastases, or poor performance status. Furthermore, elderly patients, and ethnic and racial minorities are found to be underrepresented in CRC trials [2, 3•]. Besides differences in patient populations, patients in trials receive more attention and are treated by specialized doctors in academic centers. Meanwhile,conduct of traditional RCTs to establish drug efficacy has become increasingly challenging due to identification of a growing number of predictive molecular subtypes. Trial enrollment, for instance, is challenging in rare populations such as NTRK fusion positive CRC (Fig. 1). Hence, attempts to accelerate patient access to novel drugs in case of unmet clinical need have led to authorization of therapies based on single-arm trials and surrogate end points such as response rate [4, 5]. It is recognized that all these challenges result in uncertainty regarding meaningful benefit opposed to treatment harm and societal cost once novel treatments are implemented in clinical practice. Hence, catalyzed by the digitization of healthcare, the use of RWE to complement trials has gained much interest in the oncology community.

RWE has been defined as evidence derived from analysis of RWD collected through the routine course of clinical care from a variety of sources other than traditional trials [6]. RWD on CRC is increasingly being collected in large-scale databases and registries. These provide opportunity for large population-based studies and pragmatic trials such as the registry-based RCT and studies that employ the trials-within-cohorts (TwiCs) design [7, 8]. This year, the European Organisation for Research and Treatment of Cancer (EORTC) published their position on the role of these designs in clinical cancer research [9••]. Such studies seem more representative of clinical practice due to inclusion of larger and more heterogeneous populations. Conversely, methodologic pitfalls inherent to use of RWD result in lack of trust and hesitance to base decisions solely on RWE [10]. Regulatory bodies like the US Food and Drug Administration (FDA) and the European Medicines Agency (EMA) are actively working towards further establishment of the value of RWE in supporting regulatory decision-making across all stages of drug development [11, 12].

By utilizing high-quality RWD, we believe it to be possible to learn from every patient with CRC in order to provide precision medicine to our future patients. Since RWE has globally gained attention from scientists, industry, payers, and regulators in recent years, this narrative review will provide an insight in its contributions to CRC treatment. In addition, we discuss remaining barriers and future perspectives to unlock RWE’s full potential, focusing on the medical oncology perspective.

## Using RWD for treatment effect evaluation

### Trends in population outcomes

The first cancer registries were once developed to study cancer incidence and survival and are still used for this purpose today. The identification of high-risk populations, regional disparities, and potential risk factors has been crucial for early detection and prevention of CRC. Healthcare policy decisions and initiatives are also informed by population outcome trends. For instance, in the 1990s, prognosis of CRC patients in Denmark was found to be inferior to neighboring countries. These findings led to initiation of national cancer plans and a subsequent increase in short- and long-term survival, closing the identified gap [14, 15].

Naturally, the goal of development of new oncological therapies is to improve survival while maintaining quality of life. Many population-based studies in Europe and the United States (US) have established improved survival rates for patients with CRC over the last decades [16–19]. For metastatic CRC (mCRC), median OS (mOS) in RCTs on first-line systemic treatment has almost doubled now exceeding 30 months [20, 21]. RWD on mCRC from the US Surveillance, Epidemiology and End Results (SEER) registry confirmed a meaningful increase in mOS from 12 months in 1986 to 21 months in 2015 [22•]. As the RWD availability in the SEER registry did not allow more detailed evaluation, the researchers recently conducted an additional single-center analysis and suggested increased application of liver metastasis resection, use of immunotherapy, and use of third-line chemotherapy to be the drivers behind this upward survival trend [23]. It is important to recognize that survival trends are not necessarily attributable to the accumulative effect of treatment advances alone. Mortality rates are greatly influenced by incidence rates [24], and survival may be affected by lead time bias due to earlier diagnosis after implementation of population screening and intensive follow-up programs. Also, improved diagnostic imaging leads to stage migration which influences stage stratified survival rates [25].

### Evaluation of treatment effectiveness and safety

Treatment effect can be more thoroughly evaluated using RWD sources with detailed information. While efficacy describes treatment performance in an ideal setting such as an RCT, effectiveness refers to performance in the real-world setting [26••]. The application of strict eligibility criteria in trials has led to study populations that do not resemble CRC patients in clinical practice, thereby limiting generalizability of results [3, 27]. Outcomes in systemic treatment trials are regularly found to be superior to outcomes of systemic treatments in the real world, resulting in less absolute benefit and higher levels of toxicity [28–31]. Regulatory bodies like the FDA and EMA are not responsible for ensuring that new therapies provide meaningful benefit(s), but rather that they are safe and not inferior to the standard of care. Both the American Society of Clinical Oncology (ASCO) and European Society of Medical Oncology (ESMO) have developed frameworks to assess the value of cancer therapies [32, 33]. Nevertheless, international consensus is lacking, and every country applies its own reimbursement policies [34]. Given the rapid rise of healthcare expenditure in oncology, and the realization that novel drugs do not always provide meaningful benefit [5, 35, 36], post-approval benefit-risk (re-)assessment is warranted and has led to interest in using high-quality RWD for health technology assessments (HTA) [37]. Moreover, RWD can be used to fill some of the post-registration evidence gaps with which clinicians are faced.

For instance, encorafenib-cetuximab was recently approved for pretreated patients with BRAF^V600E^ mutated mCRC following the results of the BEACON trial, which demonstrated a survival benefit of 3.4 months with a mOS of 9.3 months [38]. International treatment guidelines have since included this targeted treatment. However, despite application of strict eligibility criteria in the trial, the guideline recommendation is generalized to all patients with pretreated BRAF^V600E^ mutated mCRC, and does not elaborate on the uncertainty of benefit in patients who were not represented in the BEACON trial [39, 40]. Boccacino and colleagues found that patients treated with encorafenib-cetuximab in an Italian nominal use program had approximately 2 months shorter median OS [41]. As this nominal use program applied eligibility criteria closely resembling those of BEACON, an additional population-based study was conducted in the Netherlands which discovered that over a third of all patients treated with encorafenib-cetuximab in routine clinical care would have been ineligible for the BEACON trial [42]. These ineligible patients demonstrated significantly inferior mOS of only 6 months. Patients with a poor performance status (WHO ≥ 2) and/or symptomatic brain metastases had such a short survival time that the likelihood of meaningful benefit was deemed negligible.

We recognize that the current restrictive design of RCTs may not represent the entire patient population in which the findings will be applicable. Therefore, we agree with guideline developers’ decision to initially generalize treatment recommendations beyond the landmark trial population. However, to avoid futile or possibly even harmful treatment, we advocate that (non) randomized studies using high-quality RWD should be conducted by default to establish or refute treatment effect in populations for whom RCT evidence was not provided. This can refine selection of treatment-eligible patients to reach the eventual goal of personalized medicine. Moreover, as the efficacy-effectiveness gap is highly relevant for patient counseling, we argue that high-quality population-based effectiveness results should be incorporated in treatment guidelines [26, 43•].

RWD can also provide information on a treatment outcome or safety issue that was not assessed in the pivotal trial, including (rare) long-term safety or the understudied but highly relevant QoL measures [33, 44, 45]. This information contributes to ongoing drug safety surveillance and informs benefit-risk assessments. For example, the RECOURSE trial demonstrated a modest mOS benefit for trifluridine/tipiracil of 1.8 months compared to placebo, which led to approval and recommendation in international guidelines [36, 39]. As QoL was not assessed, a prospective evaluation of QoL and OS was performed in patients treated with trifluridine/tipiracil in routine practice using data of the Prospective Dutch Colorectal Cancer (PLCRC) cohort [46], and in a population equal to the RECOURSE trial [47, 48]. Both studies found QoL to be maintained during treatment, thus supporting trifluridine/tipiracil use in clinical practice. More recently, following trial evidence of efficacy and safety of the oral fluoropyrimidine S-1 in Western patients [49–51], additional descriptive RWE on long-term safety and cardiotoxicity recurrence supported EMA approval [52–54]. The ESMO guideline now recommends switching to S-1 in patients with mCRC who experience hand-foot syndrome or cardiovascular toxicity while being treated with capecitabine or 5-FU [39, 55].

### Comparative effectiveness research

Ideally, causal questions are answered in an RCT. This methodologic design ensures balanced patient groups with respect to both known and unknown risk factors and therefore provides the least biased evidence regarding treatment effect. RWD is increasingly used to assess causal questions, more commonly referred to as comparative effectiveness research (CER). Limitations of CER are well described and include missing data, misclassification, confounding, selection, immortal time, and treatment indication bias [56]. Treatment selection in clinical care is influenced by many characteristics including patient and physician preferences resulting in imbalanced treatment groups [57]. Advanced statistical methods are developed to correct for bias in CER, such as propensity score matching [58, 59]. Yet only variables that are measured and available for analysis can be used for such methods, which leaves the potential risk of residual confounding. There are different scenarios in which CER can be applied (Table 1). A previous publication [60••] has thoroughly described two examples of CER within the adjuvant CRC treatment setting with misleading results [61, 62]. In these cases, prior RCTs provided no evidence of treatment efficacy; however, CER performed in similar populations did suggest a treatment effect. Given that it is highly implausible that a treatment is ineffective under ideal circumstances but effective in clinical practice, CER does not provide valuable evidence in this scenario.

As discussed previously, when a landmark RCT has provided evidence of treatment efficacy, questions remain regarding treatment effect in the underreported and the trial-ineligible patient population. Pragmatic trials can be used to answer these questions; however, randomization is only considered ethical in the case of equipoise, i.e., the existence of genuine uncertainty regarding the superiority of one treatment over the other. Since treatment efficacy is most likely not limited to the trial-eligible population, performing a post-marketing pragmatic trial may be considered unethical. In this scenario, carefully designed and analyzed CER could help establish or refute treatment effectiveness in subgroups for whom RCT evidence was not provided. This can refine selection of treatment-eligible patients and reach the eventual goal of personalized medicine. Population-based CER may also inform on the overall value (benefit versus harm) of a new treatment option in clinical practice. It must be recognized that RWD are often sourced from electronic health records (eHRs) with unstandardized data. Since relevant variables—such as the experienced level of toxicity or patient performance status—are not always documented, available RWD may be of insufficient quality to yield actionable RWE.

Besides the setting in which RCT evidence is already available, CER is also performed while awaiting RCT results (Table 1). For instance, the indication for primary tumor resection (PTR) in patients with synchronous mCRC and an asymptomatic primary tumor has long been a topic of debate. Prospective evaluation was complicated due to poor acceptance of randomization by both patients and clinicians, and for a long time the only available evidence was provided by retrospective (pooled) analyses of RCTs suggesting improved survival with upfront PTR. Two propensity score–adjusted observational studies, each with a sample size greater than 10,000, were published, yet with contradictory results [63, 64]. Hence, the final answer had to be provided by prospective RCTs which have since confirmed no superiority of upfront PTR over chemotherapy alone [65, 66]. One could argue that CER results in this setting might decrease equipoise and endanger the feasibility of ongoing RCTs. Therefore, we must emphasize that quality of CER should be critically assessed, and its limitations should be acknowledged when interpreting results. Nevertheless, when effect sizes are large, and the risk of residual confounding is considered limited, CER could provide valuable and timely evidence in this scenario.

The last scenario in which CER can be conducted is the setting in which a traditional RCT is not considered ethical or feasible. For instance, when there is insufficient equipoise, or when requiring sufficient sample size or follow-up is unfeasible [67]. Randomization should, however, remain the gold standard to address causality. Hence, whenever possible, we recommend conducting pragmatic trials, such as registry-based RCTs, to optimize generalizability and answer clinical questions that are not addressed in registrational trials, e.g., optimal dosage or treatment sequence. These are recognized as efficient and cost-effective tools that combine the power of prospective randomization with the strengths of large-scale clinical registries. Such RCTs are and have been successfully conducted in CRC, examples being the RECTAL-BOOST trial in patients with locally advanced rectal cancer [68] and the MEDOCC-CrEATE trial in stage II colon cancer [69].

**Table 1:** Scenarios and the role of comparative effectiveness research (CER)

Scenario	Role CER
1. A well-designed RCT has provided evidence of no treatment efficacy	There is no role for CER
2. A well-designed RCT has provided evidence of treatment efficacy	1. Efficacy in underrepresented populations and the trial-ineligible population can remain unknown. When there is insufficient clinical equipoise, high-quality CER can evaluate efficacy in these populations 2. High-quality CER can provide valuable insight in effectiveness (for subgroups) in the real-world setting including the benefit-harm ratio, and cost-effectiveness
3. The RCT to answer a prevailing clinical question is planned or ongoing	High-quality CER could provide a timely answer to the clinical question. However, critical appraisal is imperative to prevent undoing of equipoise resulting in unfeasibility of the ongoing gold standard RCT
4. A traditional RCT is not considered ethical or feasible and will therefore not be performed	If a pragmatic randomized trial is also considered unfeasible, high-quality CER provides valuable evidence

*RCT*, randomized controlled trials

## Using RWD for precision oncology

mCRC is a heterogenous disease characterized by a fast-increasing number of distinct molecular subgroups with different prognosis and response to treatment (Fig. 1). As new drugs are being developed for rare genetic subpopulations such as patients with NTRK fusions, RET fusions, KRAS^G12C^ mutation, or ERBB2 amplified tumors, conduct of phase III RCTs has become increasingly challenging. To address unmet clinical needs, accelerated and conditional marketing approval has been introduced based on single-arm trials, pan-tumor indications, and/or surrogate endpoints. Recently, Schroder et al. successfully replicated a control arm from the IMBLAZE370 trial in mCRC using RWD, suggesting the feasibility of matched comparisons with external controls [70]. RWD is increasingly provided to regulatory bodies as context for interpretation of single-arm phase II studies [71, 72]. In 2018, Overman et al. demonstrated an encouraging 1-year OS rate of 85% for ipilimumab-nivolumab combination treatment in pretreated dMMR mCRC patients [73]. Although these single-arm results led to FDA approval, EMA approval was delayed due to lack of a control arm. RWD from the French AGEO study, the Dutch PLCRC cohort, and the US Flatiron database were analyzed demonstrating inferior survival with systemic chemotherapy [74, 75]. It is unclear from public records whether these additional data supported the regulatory approval of ipilimumab-nivolumab [76]; however, data recently presented at the ESMO annual conference demonstrates that EMA considered RWD a supportive source of efficacy- and safety-related evidence in 20% of oncology targeted drug indications from 2018 to 2022 [77].

Post-approval, RWD can be used to identify predictive biomarkers for treatment response. It was a small retrospective study which first suggested KRAS to be a negative predictive marker for cetuximab efficacy in mCRC [78], a finding that ultimately led to restriction of anti-EGFR therapy to patients with KRAS wild-type mCRC. Furthermore, a RWD discovery cohort, including whole-genome sequencing data, identified KRAS^G12^ mutations as a potential predictive biomarker for trifluridine/tipiracil resistance. Subsequently, this exploratory finding was validated in both a large real-world cohort, and in the population treated within the RECOURSE trial [79•].

## Using RWD for patient counseling

As survival results from clinical trials are not translatable to all patients in clinical practice [26••], population-based RWD currently provides more reliable estimates for subgroups of patients with comparable prognostic characteristics. Since patients prefer to discuss realistic scenarios, including best-, typical-, and worst-case median survival times [80], Hamers and colleagues recently evaluated survival scenarios for various treatment subgroups using data of over 27,000 patients with mCRC from the Netherlands Cancer Registry (NCR) [43•]. It must be emphasized that such RWE is not intended to be used to inform treatment decisions but rather to estimate patient outcomes given prevailing treatment choices. These estimations can, however, inform further care and advanced care planning, and empower patients to make informed decisions [81•].

With the goal to provide precision medicine to every patient, research efforts are increasingly focused on development of patient-level prediction models using historical data. Most prediction models provide diagnostic or prognostic probabilities using a score or risk stratification algorithm and aim to assist clinicians to identify patients who require diagnostic tests or treatment. Examples within CRC are (1) prediction tools developed to identify individuals at increased risk of CRC, which could optimize cancer screening [82], (2) models to predict recurrence of disease in the adjuvant and the oligometastatic setting, which could guide frequency of diagnostic imaging and decisions regarding postoperative adjuvant chemotherapy [83–85], and (4) models that estimate probability of survival at a specific moment in time, which could aid in decisions regarding surgery or salvage treatment [86, 87]. Nevertheless, to our knowledge, use in clinical practice is limited and there are no CRC prediction models yet that have undergone impact analysis to determine whether they indeed improve outcomes when used in clinical practice [88, 89]. Before adopting a prediction rule and evaluating impact, external validation should be performed to assess whether a prediction model is accurate and applicable to a specific setting [90]. The proportion of such external validation studies is currently small. The lack of standardization of dataset formats and variable nomenclature provides an obstacle since data curation can be very time-consuming. In the past years, external validation of several promising prediction models in the metastatic setting using population-based RWD unfortunately demonstrated suboptimal predictive performance. However, opportunities for improvement were identified for instance by including additional predictors [91, 92]. Since both the availability of high-quality RWD and methods to analyze large datasets are improving, we expect impactful prediction and decision models in the future. These could be implemented in clinical care by creating patients-like-me dashboards that can be used in the consultation room to facilitate shared decision-making.

## Using RWD to optimize treatment delivery

In addition to the development of novel therapies, the outcomes of CRC patients can also be improved by making better use of the therapies that we already have. To this end, RWD may be used to evaluate quality of care, for example, by looking at treatment adoption, guideline adherence, and access to care. In the adjuvant CRC setting, guideline adherence was previously shown to be limited [93]. Results of the IDEA trial led to the recommendation of 3 instead of 6 months of combination chemotherapy [94]. Population-based data has since demonstrated rapid implementation of these recommendations with improved guideline-concordant treatment [95]. In the metastatic setting, the evolving therapeutic landscape has led to a continuum of care in which the optimal sequence of treatment is currently unknown. A key principle is to strive to ensure that patients receive all effective agents for which they are eligible. Hence, we have used RWD to evaluate treatment patterns, practice variation, and adoption of new treatment options in the Netherlands [57, 96, 97]. These results received nationwide attention, were discussed intensively, and have resulted in practice changes. Since examples of application of RWD to evaluate care for CRC are abundant, Table 2 provides additional examples.

For interpretation of RWE, it is relevant to consider the large differences between countries in both drug access and adoption [34]. In Europe, EMA provides marketing authorization to pharmaceutical companies after assessment of drug safety and efficacy. After authorization, individual countries apply their own processes and requirements to decide on reimbursement of a registered drug. For instance, in the Czech Republic, reimbursement of regorafenib by public health insurance is or was conditional on the contribution of data to the CORECT registry with the goal to evaluate effectiveness [98]. The ongoing PROMETCO study, an international prospective longitudinal cohort, evaluates key differences between countries in the management and outcomes of mCRC [99].

## Remaining barriers and future perspectives

We have repeatedly highlighted the potential of high-quality RWE. The pursuit of actionable high-quality evidence is logical but has its challenges. Most RWE currently derives from RWD of a single center, data source, or country which strongly limits analytic possibilities. However, sharing and combining RWD is challenging for multiple reasons. First, there is the variability between RWD sources in both format and terminology. Second, a unique identifier is needed to enable proper linkage and avoid patient duplication. Third, use and sharing of RWD is protected by rather strict legal and ethical requirements to protect patient privacy and requires patient consent. It is important to realize that many patients are in favor of secondary use of their clinical data and biological samples. Hence, the EU Data Protection Regulation Recital 157 allows population-based cancer registries to operate with a “no-consent” policy under the supervision of relevant public health bodies [100]. As most countries outside the EU are not recognized to have equivalent data protection procedures in place, there is at present no practical way to share health data for research purposes, resulting in suspended and delayed international research projects [100]. Given these challenges, transparency is imperative to translate results to different settings, reproduce RWE, and compare outcomes. Reporting of RWE is however often of limited quality due to insufficiently described outcome and variable definitions, study populations, healthcare settings, and analytic procedures. To improve reporting quality of oncology RWE studies, the ESMO Real World Data and Digital Health working group developed the ESMO Guidance for Reporting Oncology real-World evidence (GROW) [101••]. These standards will not only improve the quality of reporting, but also serve as a basis for the development of study conduct assessment, and ultimately facilitate the incorporation of reliable RWE in clinical treatment guidelines.

To solve aforementioned data-related challenges, common data models (CDM) have been designed over the last decade with the aim to standardize the structure and content of observational data sources. The CDM developed by the Observational Medical Outcomes Partnership (OMOP) is recognized as most promising and is maintained and deployed by the international Observational Health Data Sciences and Informatics (OHDSI) collaboration [102, 103]. Besides transformation of registries and databases into a common format with common representation of terminology, definitions and coding scheme, this model can be used to perform systematic analyses using an open-source library of analytic procedures, which are developed by OHDSI today. We believe this will greatly aid in reproducibility, i.e., timely external validation of predictive models in different settings, and increased trust in RWE resulting from transparency. In 2022, 453 large datasets from 41 countries had been converted to the OMOP-CDM representing 12% of the world’s population [104]. As these numbers are increasing rapidly, this provides much opportunity for the near future.

Another exciting development—which relies heavily on the availability of high-quality RWD—is the application of artificial intelligence (AI) in healthcare. Machine learning (ML) and deep learning techniques are believed to have the potential to accelerate oncological drug discoveries and personalize healthcare [105•]. Multiple recent reviews have highlighted the value and current applications of AI in CRC which lies outside the scope of this review [106–108]. AI algorithms need large volumes of data to train and obtain the best results. There is a large treasure of unstructured data stored in EHRs, which is still largely unused for research. Natural language processing, a form of ML, is now able to analyze these data which could improve predictive modelling accuracy [108]. Importantly, quantity does not make up for suboptimal quality of documentation which is complicated further by lack of eHR interoperability. Hence, what is really needed is harmonization of eHRs and arranging them to serve not only clinical but also research purposes. Many countries aim to establish one country-wide eHR system with comprehensive sharing of records from multiple providers [109]. To overcome the challenge regarding patient privacy, multiple observational research groups are working on a privacy-by-design approach using federated learning; a ML technique that performs an analysis across multiple decentralized data sources [110•]. The aggregated outcomes and model parameters from the decentralized sources are combined in a central server that provides the researcher with one result based on complex mathematics. This method does not require exchanging of raw and sensitive patient data. Although AI techniques are promising and start to impact diagnostic imaging in clinical practice, application in CRC treatment is still in the experimental stage and faces many challenges. Most important to realize is that they are not yet able to make accurate causal inference and are therefore not equipped to recommend the optimal treatment for an individual CRC patient [105•].

To conclude, in the trial design phase, we recommend to carefully consider pragmatic trial designs to increase generalizability whenever suitable; in the drug regulatory phase to provide conditional marketing approval for treatment of patients who would have been ineligible for the registrational trial—awaiting planned evaluation of outcomes in the real-world; and lastly regarding the clinical application, effectiveness results of high-quality RWE studies should be incorporated in treatment guidelines to support optimal patient counseling. We emphasize that both RWD and results from RCTs are needed to improve care for patients with CRC. A coordinated effort among all stakeholders, i.e., healthcare professionals, patient advocates, HTA bodies and payers, regulators, epidemiologists, and statisticians, is needed to achieve high-quality primary data and ensure high-quality secondary use. Supported by further sophistication of sources and analytical methods, we believe it to be possible to use RWD to answer questions of all stakeholders, reduce oncological healthcare costs, and, most importantly, improve patient care and outcomes.

**Table 2:** Illustrative examples of RWD used for treatment delivery optimization

Subject	Objective	Data	Key findings	Ref
*Treatment adherence by patients*	Assess adherence and duration of treatment with trifluridine/tipiracil versus regorafenib	469 trifluridine/tipiracil and 311 regorafenib users from the QVIA Real-World Data Adjudicated Claims U.S. database (Oct 2014-Jul 2017)	Trifluridine/tipiracil use was associated with higher medication adherence and longer time to discontinuation compared to regorafenib	[111]
*Guideline adherence*	Determine rates of and factors associated with adherence to the NCCN treatment guideline for colon cancer	Data of the National Cancer Database including 173.243 patients treated for colon cancer (2003–2007)	Among patients with high-risk stage II or stage III disease, older patients with pre-existing comorbidities and patients with lower socioeconomic status were less likely to be offered adjuvant chemotherapy	[93]
*Practice variation*	Evaluate treatment patterns and associated variables in the systemic treatment of mCRC in the Netherlands	Random sample of 2222 mCRC patients diagnosed in 20 hospitals from 2008 to 2015 from the NCR	Significant inter-hospital variation was identified in targeted therapy administration, which may effect outcome. The data suggests that practice variation is based on individual strategy of hospitals rather than guideline recommendations or patient-driven decisions	[97]
*Treatment underutilization*	Evaluate the adoption rate of FOLFOXIRI-B in patients with mCRC and investigate the perspectives of medical oncologists towards this treatment option	Within 1 study week, data were retrieved from eHRs of 47 hospitals on all patients with mCRC referred between Nov 2020 and Jan 2021, *n* = 402. Interviews were conducted with 101 medical oncologists from 52 hospitals	Prescription rates marginally increased in 5 years. Most medical oncologists discuss FOLFOXIRI-B but communicate a preference for doublet chemotherapy to patients. These oncologists reported a significantly lower awareness of guidelines and trial results	[57]
*Treatment overutilization*	Evaluate the use of radiotherapy (RT) in the Netherlands and discuss Dutch practice in the context of current literature	Data from the Dutch Surgical Colorectal Audit (DSCA) on 6784 patients surgically treated for primary rectal cancer in 2009–2011	From a European perspective, a high percentage of rectal cancer patients are treated with RT in the Netherlands. Considerable hospital variation was observed for RT in stage I and the proportion of chemoradiotherapy among all RT schemes	[112]
*Treatment patterns*	Describe the treatment of metachronous colorectal cancer metastases in a population-based cohort	5412 patients with stage I–III CRC diagnosed between Jan and Jun 2015 who were surgically treated with curative intent selected from the NCR	Patients with metastases confined to the liver and lung have the highest rates of local treatment (LT). The number of patients who underwent LT is higher than previously reported	[113]
*Non-participation in screening program*	Investigate differences between participants and non-participants in the Dutch CRC screening program	Data from the Dutch screening system linked with demographic characteristics from Statistics Netherlands including all invitees to the Dutch CRC screening program in 2018 and 2019	Individuals who were single, with a migration background or low income, were the least likely to participate	[114]
*Subgroup identification for molecular testing*	Estimate the prevalence of NTRK fusions in microsatellite instable (MSI) mCRC and determine patient characteristics and clinical behavior of this NTRK fusion subtype	FFPE tissue was requested from all (*n* = 203) MSI mCRC patients diagnosed between 2015 and 2020 in the Netherlands	NTRK fusions are most prevalent (23%) in RAS and BRAF wild-type MSI mCRC patients. Benefit from chemotherapy or anti-EGFR therapy in NTRK fusion positive patients was limited, but all immunotherapy-treated patients had ongoing response	[115]
*Management and outcomes in elderly patients*	Describe management and outcomes of surgical resection of CRC liver metastases (LM) in elderly patients in routine practice	Data of the Ontario Cancer Registry on 1310 patients who underwent resection of LM between Jan 2002 and Dec 2009, classified into age groups	Resection of CRC LM is associated with greater risk of postoperative mortality among elderly patients despite less aggressive treatment. Although the long-term outcomes are inferior to younger patients, a substantial proportion of elderly patients will have long-term survival	[116]
*Racial differences*	Evaluate disparity in receipt of adjuvant chemotherapy	Patients diagnosed with stage III colon cancer were randomly sampled from the SEER program from the years 1990, 1991, 1995, 2000, 2005, and 2010	There were marked racial disparities in the time period of 1990 to 1991 and again in 2010, with black patients less likely to receive adjuvant chemotherapy as compared with white patients	[117]

*NCCN*, National Comprehensive Cancer Network; *NCR*, Netherlands Cancer Registry; *FFPE*, Formalin-fixed paraffin-embedded
